# A Rare but Life-Threatening Case of Spontaneous Hemopneumothorax in a Young Male

**DOI:** 10.7759/cureus.49715

**Published:** 2023-11-30

**Authors:** Shamayel Almulhem, Rakan Mounla Ali

**Affiliations:** 1 College of Medicine, King Saud bin Abdulaziz University for Health Sciences, King Abdullah International Medical Research Center, Riyadh, SAU; 2 Thoracic Surgery, King Abdulaziz Medical City Riyadh, Riyadh, SAU

**Keywords:** pleural adhesion, apical pulmonary bleb, hemodynamic instability, pulmonary bleb wedge resection, chest pain, emergency room, chest tube, respiratory distress, video-assisted thoracoscopic surgery, hemopneumothorax

## Abstract

Spontaneous hemopneumothorax (SHP) is the spontaneous accumulation of both blood and air within the pleural space without any previous medical or trauma history. Despite its rare existence, it is one of the most life-threatening conditions seen in the emergency department. Even though SHP is an uncommon presentation, early recognition and prompt intervention are essential because of its rapidly deteriorating nature leading to respiratory distress and hemodynamic instability. This is a case of a 20-year-old male who presented in the emergency room complaining of sudden chest pain and respiratory distress. After physical examination and radiological investigation, a diagnosis of left-sided spontaneous hemopneumothorax was the top differential that consisted of the patient’s presentation and chest X-ray. Subsequently, a chest tube was inserted to drain the blood in the pleural cavity. In addition to the presence of blood in the chest tube, the serum hemoglobin levels of the patient were low, which suggested the diagnosis of hemopneumothorax. After that, the patient underwent video-assisted thoracoscopic surgery (VATS) which demonstrated a significant amount of pleural blood clots, pleural adhesions, and apical blebs in the lung. Through this procedure, the source of the bleeding was found to be a ruptured adhesion in the left lung. By the end of this surgical intervention, the adhesions were cauterized and the blebs were resected. Post-operatively, the patient stabilized and had a full lung expansion upon follow-up. This case emphasizes the importance of early recognition, diagnosis, and prompt surgical management of SPH to prevent life-threatening complications.

## Introduction

Spontaneous hemopneumothorax (SHP) is the spontaneous accumulation of both blood and air within the pleural space without any history of trauma or specific cause [1,2]. SHP is a rare thoracic disease with an incidence of 0.5-12% [1-3]. Unlike spontaneous pneumothorax, SHP is an uncommon condition that is life-threatening if the bleeding is massive. SHP must be excluded in any case presenting with rapid respiratory collapse and a sudden onset of unexplained hemodynamic instability. Although it is a surgical emergency, early recognition is often delayed by healthcare providers due to the decreased awareness of this rare condition [3,4]. This study reports a case of spontaneous hemopneumothorax that was treated promptly with a chest tube, followed by video-assisted thoracoscopic surgery (VATS).

## Case presentation

A 20-year-old male presented in the emergency room (ER) at King Abdulaziz Medical City with a one-day history of sudden left-sided chest pain that was pleuritic in nature and increased upon breathing and movement. The patient's symptoms were rapidly deteriorating. The patient is an active smoker with a history of asthma. The patient did not complain of cough or loss of consciousness and he was afebrile. There was no recent history of trauma, surgical interventions, or medication use, nor was there any previous history of similar complaints. Upon arrival, his blood pressure was 93/51 mmHg. His heart rate was 130 beats/minute. His respiratory rate was 26 breaths/minute with an O_2_ saturation of 93% and his core body temperature was 36°C.

**Figure 1:**
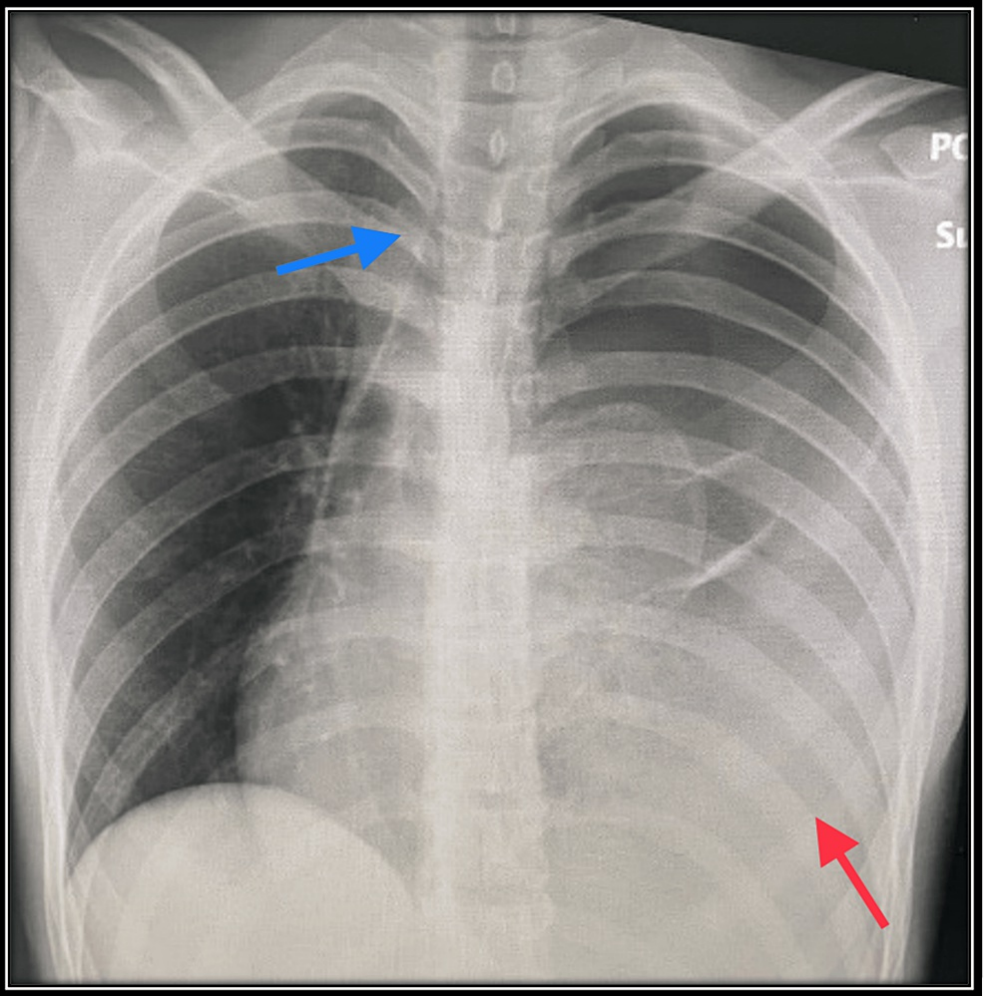
Chest X-ray of the patient on arrival at ER. The radiographic findings of a supine chest X-ray revealed a left-sided primary spontaneous pneumothorax and hemothorax (red arrow) with tracheal deviation to the contralateral side (blue arrow).

Upon arrival at the ER, the patient was conscious, tachypneic, tachycardiac, and in moderate respiratory distress. Upon lung auscultation, there was decreased air entry in the left side of the chest. Based on physical examination findings, pneumothorax was suspected, so a chest X-ray (CXR) was ordered (Figure 1). The radiographic findings showed left-sided spontaneous hemopneumothorax.

**Figure 2:**
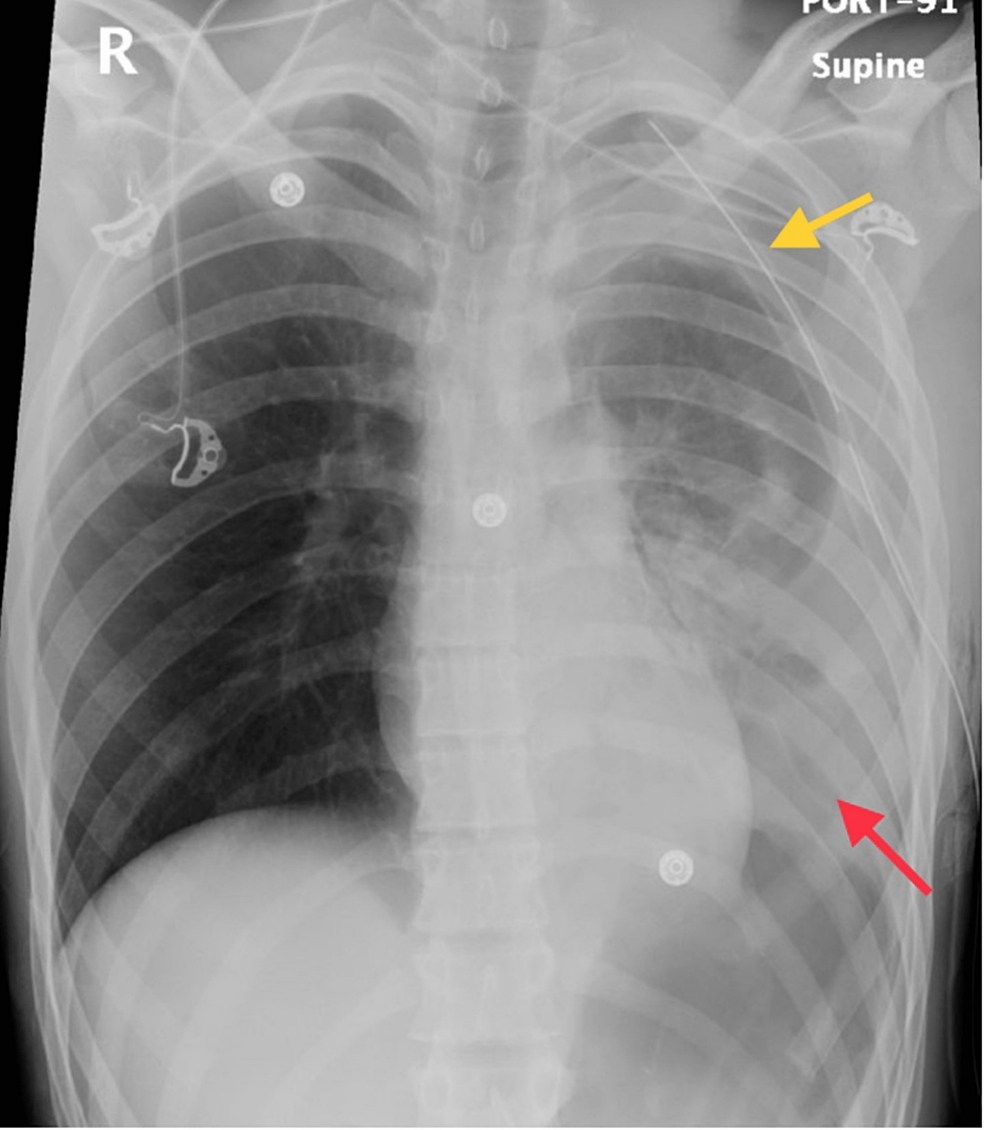
A repeated chest X-ray was performed to evaluate the position of the chest tube (yellow arrow). The image shows blood residuals on the left side (red arrow).

Ketamine was administered through an intravenous line with a dose of 50 mg and local anesthesia was applied as a preparation for chest tube insertion. A chest tube with a size of 28 French was inserted in the fifth intercostal space anterior midaxillary line under aseptic techniques. Upon insertion of the chest tube in the chest cavity, fresh blood was spontaneously drained out of the tube (Figure 2).

**Figure 3:**
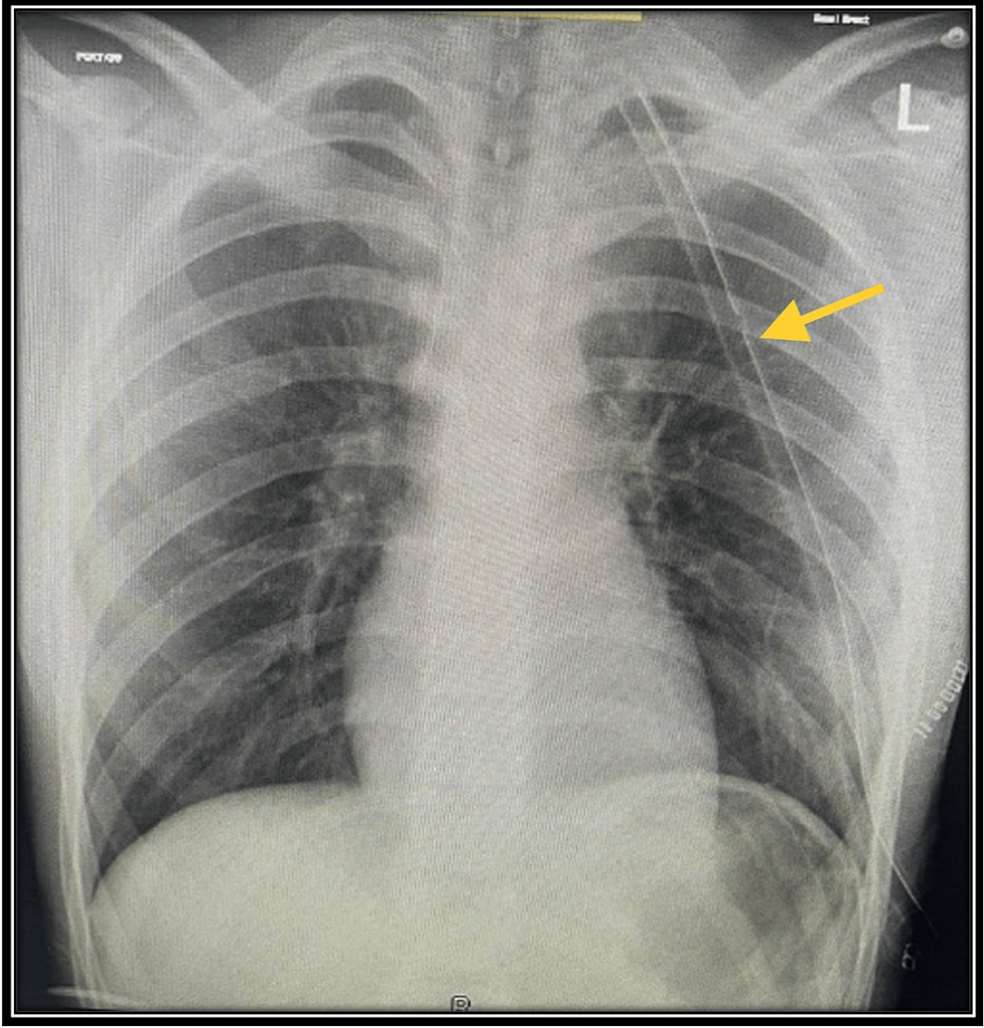
Chest X-ray after surgery showed resolution of hemopneumothorax with fully expanded lung (yellow arrow is the chest tube).

The initial volume upon insertion of the chest tube reached 1500 mL. The level of his serum hemoglobin was 10.6 g/dL. The patient was immediately resuscitated with one liter of crystalloid fluid. He was subsequently evaluated by the thoracic surgical team and taken for an emergent video-assisted thoracoscopy surgery (VATS). Emergency surgery was done. VATS was done through a minimally invasive endoscopic surgical procedure that required the insertion of an endoscope through the thoracic wall (thoracoscopy). Upon thoracoscopy, a large amount of blood clots were observed, and vascularized apical pleural adhesions with multiple blebs at the apex of the lung were also identified. The primary source of bleeding was most likely to be secondary to the ruptured adhesion. Hemostasis was achieved via cauterization of the bleeding adhesions and wedge resection of the apical blebs. A sample was taken for a histopathology examination of the resected bleb which showed lung parenchyma with emphysematous change and hemorrhage, pleura with fibrosis, and reactive mesothelial hyperplasia which is consistent with lung bullae. The patient remained stable post-operatively and had minimal drainage from the chest tube with no air leak (Figure 3).

Subsequently, the chest tube was removed on the fifth post-operative day. The patient was stable and discharged on the same day. Seven days after hospital discharge, the patient came for a follow-up asymptotically, and a CXR confirmed full lung expansion. To check for any recurrence of SHP, the patient was seen again after six months by the thoracic clinic which confirmed the lack of recurrence.

## Discussion

Spontaneous hemopneumothorax (SHP) was first reported by Läennec in 1829 from an autopsy [5]. Despite the well-documented nature of this condition in the literature, SHP is rarely encountered in clinical practice. The definition of SHP is still not consistent, as some authors defined it as blood accumulation of more than 400 mL within the pleural cavity in the case of primary spontaneous pneumothorax [6]. However, a meta-analysis done in Iraq exploring 35 studies that included 358 patients suggested a more accurate definition, which was the presence of any amount of blood drained in a case of known spontaneous pneumothorax. The latter definition is consistent with the cases reported in the literature since there were multiple cases reported that the initial amount of blood drained was less than 400 mL [6].

Bleeding in SHP cases is most commonly due to three proposed mechanisms. First, a torn adhesion band between the parietal and visceral pleura resulting from a collapsed lung. Second, a rupture of both the vascularized bulla and the underlying lung parenchyma. Third, torn congenital aberrant vessels branching from the plural cupula that are distributed in and around the bullae in the apex of the lung [6,5]. The development of SHP was shown in the literature to have gender differences. Chen et al. stated that there is a 30 times higher male predominance in the development of SHP [7]. A couple of papers reported that the development of SHP was almost always secondary to primary spontaneous pneumothorax (PSP) [3,4]. PSP presents in patients without any underlying lung disease, with 81-90% of patients having subpleural bullae identified on CT or at surgical exploration [3]. One risk factor for PSP development is cigarette smoking. Cheng et al. demonstrated that cigarette smoking increases the relative risk of PSP development by nine-fold in women and 22-fold in males [2]. However, a meta-analysis revealed that smoking was reported less in SHP in comparison to PSP (33% and 90%, respectively) [8]. Yet smoking history was seen in the current case and other SHP cases reported in the literature [7-9].

The most commonly encountered presenting symptoms are chest pain (98.8%) and shortness of breath (67.8%) [8]. Even though these symptoms are commonly seen in PSP as well, the possibility of developing hypovolemic shock and progressing to rapid clinical deterioration is what distinguishes SHP from PSP, as shown in the current case and a variety of other case reports in the literature [2,3,8]. Kakamad et al. mentioned that among 358 patients diagnosed with SHP, 35.8% presented with a shock state during admission [8].

The most useful tool for SHP diagnosis is the upright/setting chest X-ray. The typical picture of hemopneumothorax is associated with the presence of an air-fluid level that obscures the costophrenic angle. However, up to 10% of the CXR may only show simple pneumothorax if the diagnostic testing is done earlier with the radiological evidence of hemothorax appearing later on. Consequently, clinicians should consider SHP as one of the differentials in any patient presenting initially with simple pneumothorax. Despite the fact that CT is the most sensitive investigation for SHP, CT is usually unnecessary in the acute setting unless the diagnosis of SHP is in doubt or excludes secondary causes [10].

Once SHP is diagnosed, fluid resuscitation followed by a chest tube should be done initially. SHP can be managed conservatively if the bleeding subsides within 24 hours or if the patient is deemed hemodynamically stable [6,10]. Nevertheless, performing VATS may be more viable in some circumstances. Such circumstances include the rapid development of clinical deterioration, impaired lung expansion from blood clots collecting in the pleural cavity, and the development of rebleeding from attached blood clots on the lung surface. Therefore, when compared to the conventional thoracotomy procedure which is more invasive, patients with SHP should immediately undergo surgical management by VATS due to the associated complications seen if the patient is treated conservatively [10-13].

One study showed that early surgical intervention by VATS decreased the incidence of delayed surgical exploration and decortication. The main indication for surgery is a patient with signs of shock and continuous bleeding greater than 100 mL/hour. In comparison to conventional thoracotomy, VATS is superior due to less post-operative pain, a shorter hospital stay, and better recovery of lung function. In the current case, initial fluid resuscitation was initiated, followed by the insertion of a chest tube, and early VATS intervention was performed as recommended by recent medical literature [10,11,13]. However, there are no therapeutic guidelines yet on when to intervene surgically.

## Conclusions

This is a rare disease entity to be identified in emergency situations. Despite the rarity of SHP, the consequences are devastating. Therefore, SHP should be suspected in any patient who is a young male presenting with a sudden onset of chest pain and shortness of breath with or without signs of shock as well as classical hemopneumothorax chest X-ray findings. Early recognition and surgical intervention due to the potentially life-threatening consequences are critical. Certainly, conservative management is valuable as a bridge to stabilize the patient before undergoing VATS.
